# Re-irradiation of a Classic Kaposi’s Sarcoma Using Volumetric Modulated Arc Therapy

**DOI:** 10.7759/cureus.51782

**Published:** 2024-01-07

**Authors:** Zhe Chen, Steven Daveluy, Geoff Baran, Michael Joiner, Steven Miller

**Affiliations:** 1 Radiation Oncology, Wayne State University School of Medicine, Detroit, USA; 2 Dermatology, Wayne State University School of Medicine, Detroit, USA

**Keywords:** re-irradiation, volumetric-modulated arc therapy, clinical dermatology, radiation and clinical oncology, kaposi sarcoma hiv negative

## Abstract

A black male in his 60s diagnosed with classic Kaposi’s sarcoma presented with multiple cutaneous nodules and edema of the right foot and lower leg. He was initially treated with alitretinoin 1% topical treatment. However, 16 months after treatment with the alitretinoin, the skin lesions progressed, and he subsequently underwent a course of radiation therapy to a total dose of 2000 centigrays (cGy) in five fractions to his right foot and lower extremities. Approximately 1.5 years after the radiation therapy was completed, multiple new lesions developed on the right foot and distal lower leg. He then underwent a course of re-irradiation to this area using volumetric modulated arc therapy (VMAT) to a total dose of 3300 cGy in 11 fractions. At a four-week follow-up visit, the skin lesions had completely resolved; however, the patient experienced mild edema and tenderness of the right foot and lower leg. Although long-term outcomes need to be followed, re-irradiation showed positive short-term outcomes for classic Kaposi's sarcoma.

## Introduction

Kaposi’s sarcoma is a low-grade vasculoendothelial malignancy associated with human herpes virus-8 (HHV-8). Kaposi’s sarcoma classically affects the lower extremities in older males and often presents as single or multiple skin lesion(s) with or without mucosal, visceral, or nodal involvement. These lesions may cause pain, bleeding, ulceration, infection, or lymphedema [1].

Kaposi’s sarcoma is neither a curable nor a life-threatening disorder. Classic Kaposi’s sarcoma is usually indolent and treatable for palliative and or cosmetic purposes. Individualized treatment strategies are based on the location and extent of the lesions, risk of tumor-related complications from treatment, patients’ immunovirological status, and systemic diseases. For limited cutaneous lesions, treatments typically include topical agents, local excision, radiotherapy, cryotherapy, intralesional injections, or a combination of these treatments. Systemic therapy (chemotherapy and immunomodulatory agents) may be used for disseminated, visceral, and nodal diseases. For recurrent or progressive lesions, the use of previously effective treatments should be considered [2].

## Case presentation

**Figure 1:**
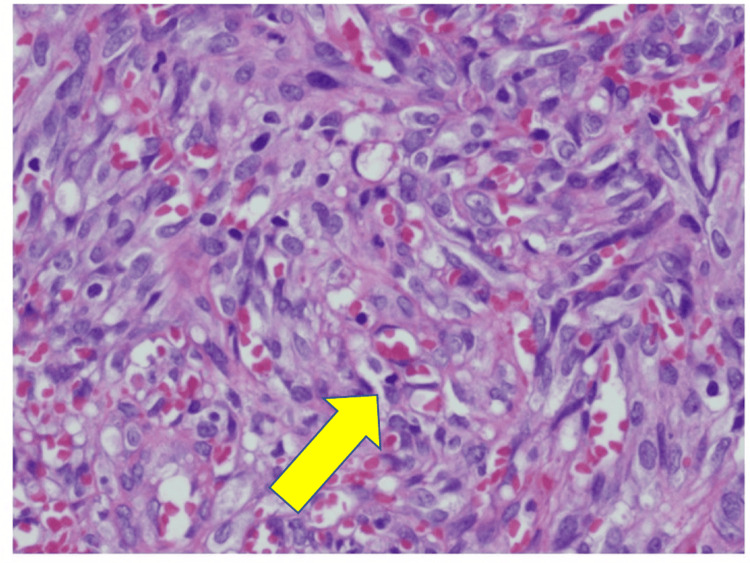
200X magnification. Hematoxylin and eosin stains show ill-defined, interweaving vascular channels. Immunohistochemistry showed that the lesional cells were positive for HHV and endothelial markers CD34 and D2-40. HHV: Human herpes virus.

The patient was a black male in his 60s who initially presented with two purple papules on the right medial foot and the plantar surface of the second toe. The biopsy was consistent with Kaposi’s sarcoma, with immunohistochemistry confirming HHV8 (Figure 1).

**Figure 2:**
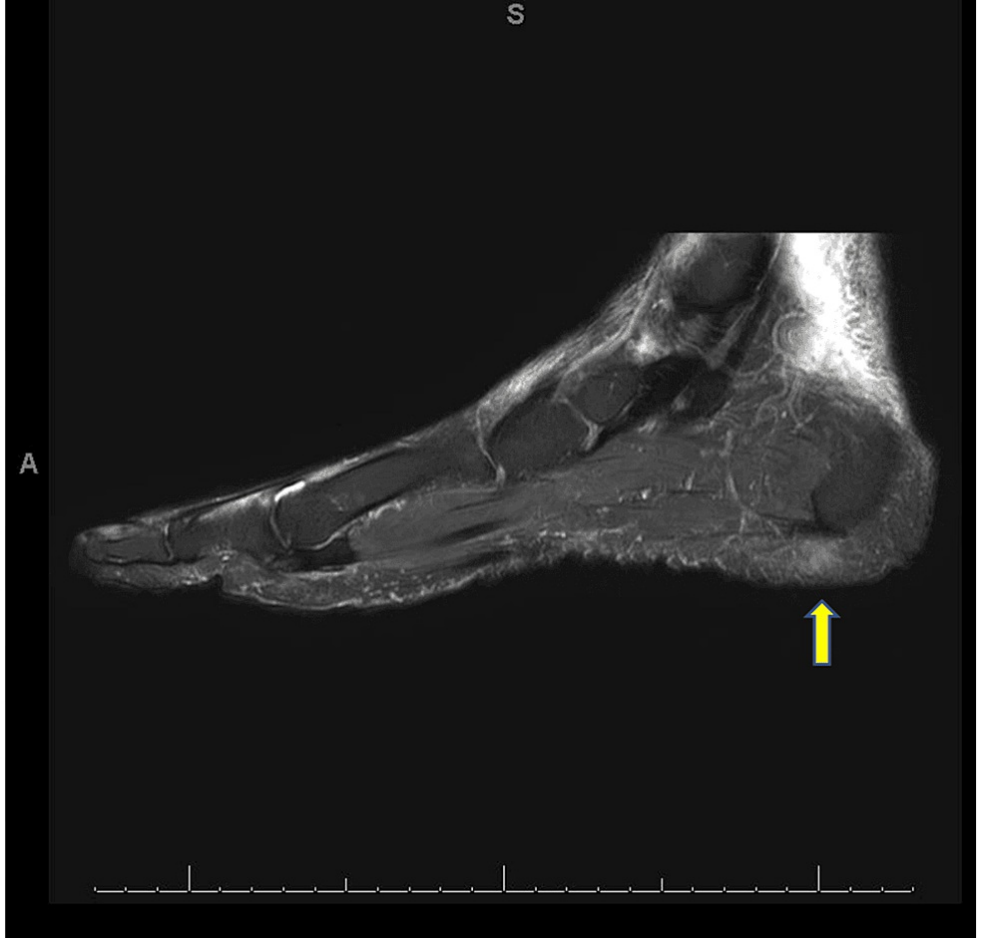
Right medial foot lesion

**Figure 3:**
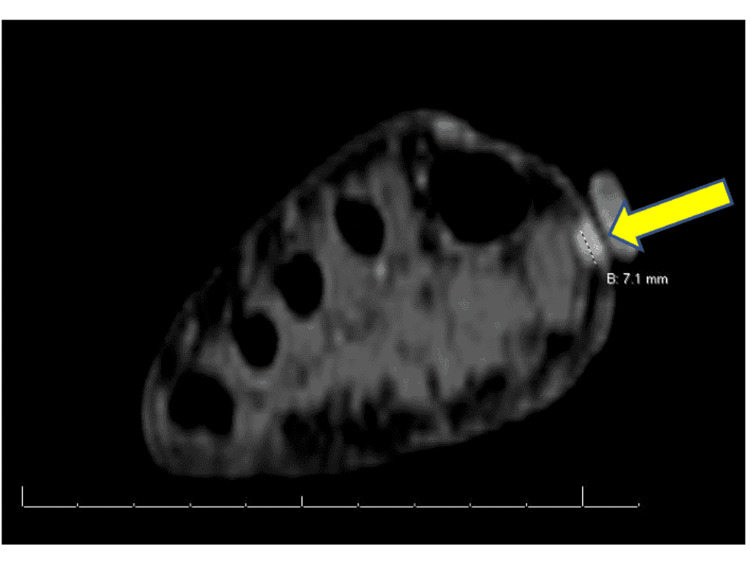
Right toe lesion

The HIV test was negative, and an MRI of the right foot revealed a 0.6 × 0.7 × 0.8 cm enhancing subcutaneous nodule along the right medial foot (Figure 2) and a 0.4 × 0.7 × 0.7 cm subtle subcutaneous enhancement along the plantar surface of the second toe (Figure 3).

The patient had a past medical history remarkable for hypertension, diabetes mellitus, and one cardiac catheterization. His family history was unremarkable. He denied any history of systemic corticosteroid use. He was initially prescribed alitretinoin 1% solution twice daily with resolution of lesions after nine months. However, seven months later, several small purple nodules developed on the medial and plantar area of the right foot with marked edema up to the midcalf.

**Figure 4:**
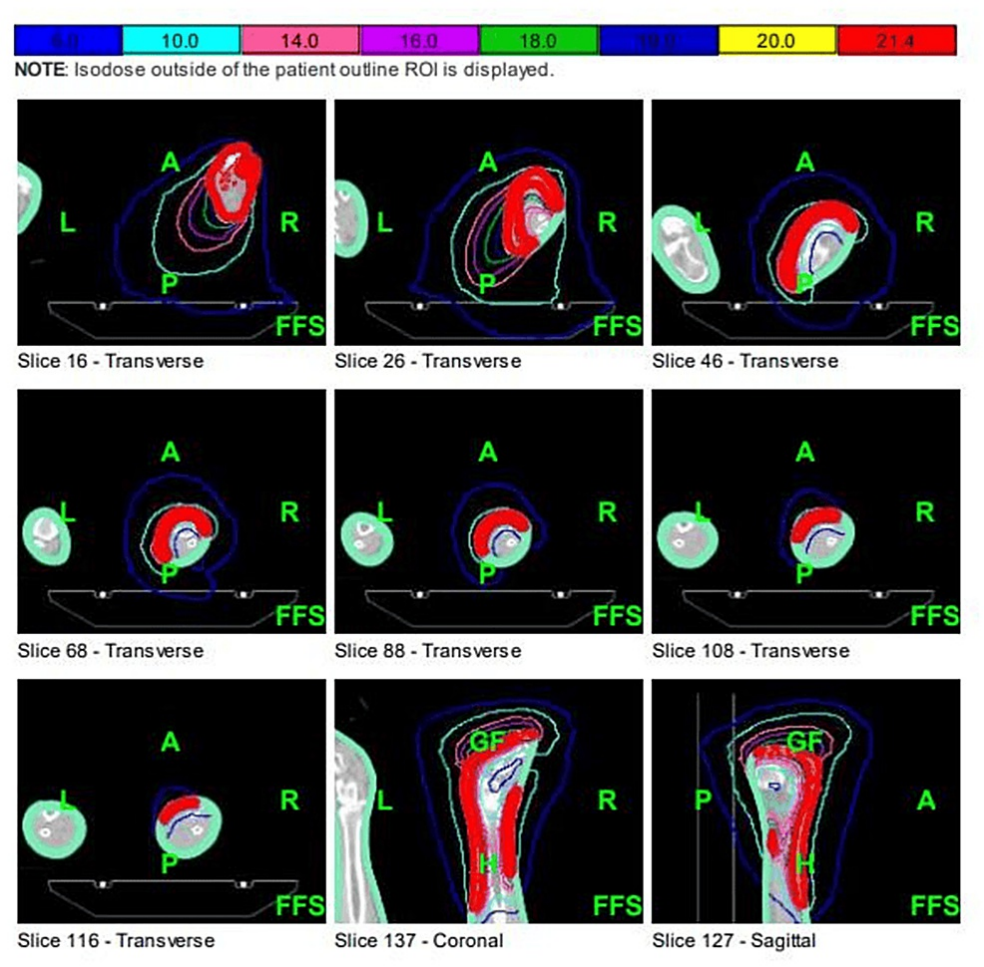
Initial radiation therapy treatment

He underwent a course of radiation therapy using 6 Megavolt (MV) x-rays using intensity-modulated radiation therapy (IMRT) to a total dose of 2000 cGy in five fractions via a tomotherapy linear accelerator. The clinical target volume included the skin of the entire right foot and the medial aspect of the right ankle up to an approximate depth of 0.5 cm (Figure 4).

A four-week follow-up evaluation revealed mild erythema and discoloration of the right foot with mild tenderness and less than 1+ pitting edema. A three-month follow-up revealed that the lesions had completely resolved with some mild hyperpigmentation of the skin of the treatment area, but his right lower extremity swelling had slightly worsened.

**Figure 5:**
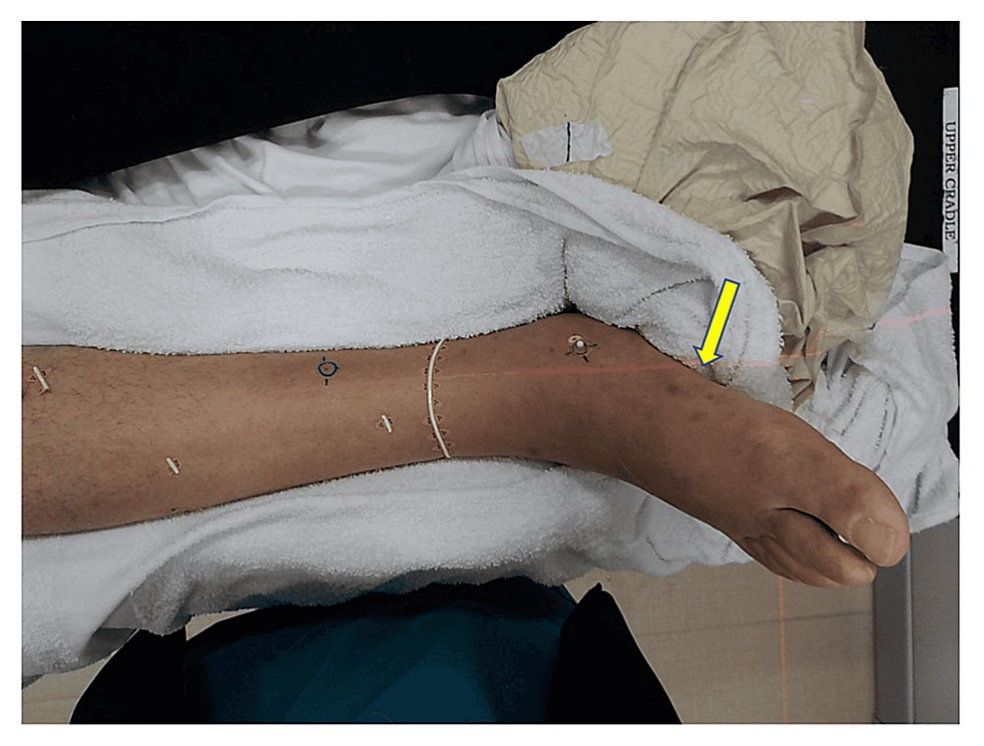
Pre-treatment images of the right foot; note the areas of discoloration involving the medial foot

A follow-up examination at month 16 revealed several new small purple papules involving the right foot and lower leg, which were inside and outside of the previous radiation treatment fields. He also noted mild edema involving the right lower leg. Timolol 0.5% ophthalmic solution twice daily was started in combination with alitretinoin solution. The lesions improved following topical treatment. However, 10 months later, one of the lesions on the right lateral foot presented as a 6-mm bleeding-eroded papule. Several small purple papules/nodules on the right foot and 2+ pitting edema on the right lower extremity were also observed (Figure 5).

**Figure 6:**
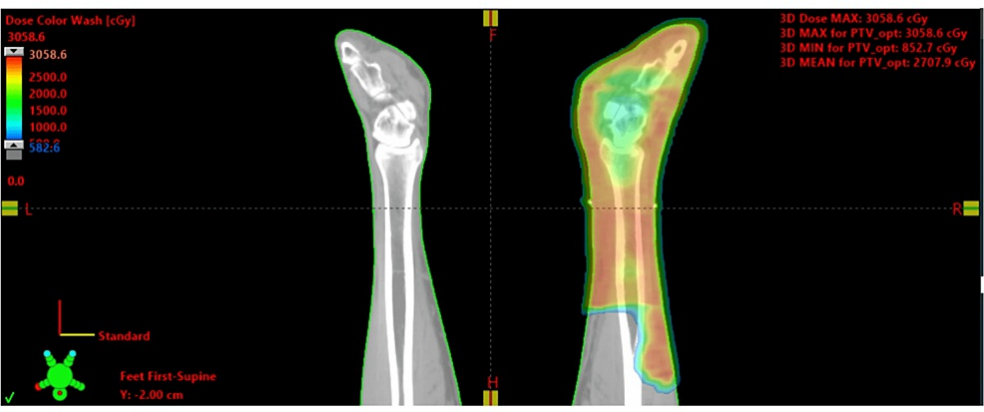
Radiation dose color wash of the right lower leg planned using volumetric modulated arc therapy (VMAT)

Treatment options were discussed with the patient who elected to undergo re-irradiation to the right lower leg and foot. He was treated with a course of radiation therapy using 6MV VMAT to a total dose of 3300 cGy in 11 fractions using a Varian IX linear accelerator, with the treatment volume including the skin of the right foot, ankle, and distal right leg to a depth of 0.5 cm (Figure 6). The equivalent dose in 2Gy fractions (EQD2) for the treatment of this patient is as follows: 28 Gy for 4 Gy x 5 fractions and 39.6 Gy for 300 cGy x 11 fractions (assuming an alpha/beta of 3 for a sarcoma). The total EQD2 from both treatments would be 67.6 Gy.

He tolerated the treatment well overall, with no significant treatment breaks. He developed the expected radiation side effects including dry and moist desquamation involving the right lower extremity, which was treated conservatively with Aquaphor and Silvadene cream.

At a four-week follow-up after re-irradiation, an examination of the right lower extremity and foot revealed hyperpigmentation, dry desquamation, and improved edema. He underwent a four-month follow-up evaluation, and at the time, the desquamation and swelling of the right lower extremity had significantly improved with no pitting edema of the right lower extremity. The Kaposi’s sarcoma lesions had disappeared with only minimal hyperpigmentation of the right lower extremity. He was able to walk comfortably and wear shoes, which he had not been able to do previously because of the edema.

## Discussion

Radiation therapy (RT) is commonly used for local Kaposi’s sarcoma treatment due to its high radiosensitivity and response rate [3-6]. Electrons and low-energy photons are commonly used for Kaposi’s sarcoma RT, and bolus materials (e.g., water equivalent material, wax, etc.) are frequently applied to provide adequate dose coverage on the skin surface and a homogeneous dose distribution for irregular surfaces (e.g., extremities) [7]. Electron beam therapy can effectively spare normal tissue due to its limited penetration depth. However, it may require a large number of treatment fields when treating multiple lesions with complex target geometry (e.g., foot) or a large treatment field. This can result in long treatment times, overlap, and underlap of treatment fields at the field junctions, which can create areas of overdose and underdose. On the other hand, VMAT delivers radiation to a target volume using a linear accelerator, a multi-leaf collimator, and one or more continuously rotating gantry arc(s). The shape, intensity, and direction of the radiation field are modulated throughout the treatment creating a dose cloud that is very conformal to the target volume while sparing the organs at risk (OARs). VMAT has also demonstrated improved dose distribution (better target conformity and normal tissue sparing) and treatment efficiency (less treatment time and labor) for multitarget treatments as compared to electron beam therapy, intensity-modulated RT (IMRT), and high-dose-rate (HDR) brachytherapy [8,9].

Although there is no consensus on the optimal dose and fractionation regimen, treatment decisions should be made based on the patient’s symptoms, medical condition, life expectancy, location and extent of the lesions, and relevant previous treatments [10,11]. An 8 Gray (Gy) single-fraction treatment has been recommended to treat patients with Kaposi’s sarcoma with a limited life expectancy as it provides a satisfactory palliative response (~80%) with a limited duration (relapse in four to seven months post-RT) [12-15]. For long-term local control, doses greater than 20 Gy in multifractions have demonstrated a higher response rate (>90%) with minimal toxicities [7,16-18]. A randomized study demonstrated no significant differences in treatment response, local control, and acute/late toxicities between 20 Gy in five fractions and 24 Gy in 12 fractions for endemic Kaposi’s sarcoma. However, caution should be exercised for hypofractionated RT as it may cause severe toxicities for patients with extensive lesions and/or severe lymphoedema (four sites at 20 Gy vs. one site at 24 Gy developed ulceration or necrosis) [19]. Stelzer and Griffin compared 8 Gy in a single-fraction, 20 Gy in 10 fractions, and 40 Gy in 20 fractions for endemic Kaposi’s sarcoma and reported that fractionated higher total radiation dose may improve tumor response (complete response rate: 8 Gy 30%, 20 Gy 79%, and 40 Gy 83%) and local control (median time to failure: 8 Gy 13 weeks, 20 Gy 26 weeks, and 40 Gy 43 weeks) [12]. However, acute and late toxicity rates could increase with increasing total radiation dose. Several studies also recommended the use of 30 Gy in 10 or 15 fractions with a high response rate (>90%) and limited toxicities (e.g., erythema, dry desquamation, and hyperpigmentation) [16,17,20].

Due to the nature of Kaposi’s sarcoma, recurrence and progression often occur. However, there are only a few studies that mention RT retreatment. Stelzer and Griffin presented seven recurrent Kaposi's sarcoma lesions that were initially treated with electron therapy. The patients were treated with re-irradiation to the recurrent lesion to a dose of 8 Gy in one fraction, 20 Gy in 10 fractions, or 14 Gy in seven fractions. Six of the patients treated had a complete response, and one patient had a partial response [12]. Alternatively, Tsao et al. reported 18 progressive Kaposi's sarcoma lesions that were initially treated with external beam RT using doses ranging from 6 Gy in one fraction to 30 Gy in 10 fractions that all underwent re-irradiation. Two patients were lost to follow-up, and zero of the remaining 16 lesions showed a complete response; 50% showed a partial response, and 50% showed continued progression [21]. However, there was a lack of clarity about the re-irradiation dose used in these two studies. Another single institution study evaluated 19 progressive Kaposi's sarcoma lesions that were re-irradiated with the following fractionation schemes: 8 Gy single-fraction or 20 Gy in 10 fractions. Of those patients, 53% achieved complete response, and 47% had stable disease [22].

RT is very well tolerated for the treatment of Kaposi’s sarcoma and is usually accompanied by only mild toxicities (e.g., erythema of the skin, alopecia, hyperpigmentation, edema, etc.). However, the major concern with re-irradiation is the higher probability of severe toxicities (ulceration or necrosis of the skin) secondary to the total combined radiation dose. In this case, VMAT with the approximate total dose of 3300 cGy in 300 cGy per fraction was utilized for re-irradiation treatment of multicentric lesions on the right foot and lower leg. The Kaposi's sarcoma lesions completely resolved; however, there was treatment associated with 1-2 + right lower leg edema. Only mild acute skin toxicity was observed after re-irradiation. Follow-ups are needed to assess late toxicities and local control status.

This is the first report regarding the re-irradiation treatment plan for Kaposi’s sarcoma using the VMAT technique to deliver a total dose of 3300 cGy in 11 fractions. The patient received a partial response for four weeks and a complete response at his two-month follow-up. The efficacy of this treatment will be further evaluated.

## Conclusions

Classic Kaposi’s sarcoma with multicentric cutaneous lesions and edema of the extremities previously treated with external beam irradiation can be safely retreated. This can be accomplished with the use of VMAT. With VMAT, the complex treatment volume can be more adequate with the prescribed dose of radiation with a significant decrease in dose to the OARs. Further studies need to be performed to better evaluate this treatment.
